# Effect of *Tinospora crispa* on thioacetamide-induced liver cirrhosis in rats

**DOI:** 10.4103/0253-7613.75673

**Published:** 2011-02

**Authors:** Farkaad A. Kadir, Faizah Othman, Mahmood Ameen Abdulla, Farida Hussan, Pouya Hassandarvish

**Affiliations:** Department of Anatomy, Faculty of Medicine, Universiti Kebangsaan Malaysia, Kuala Lumpur, Malaysia; 1 Department of Molecular Medicine, Faculty of Medicine, Universiti Malaya, Kuala Lumpur, Malaysia

**Keywords:** Antioxidant, ethanol extract, liver cirrhosis, *Tinospora crispa*, traditional medicine

## Abstract

**Objectives:**

This study was conducted to determine the effect of ethanolic extract of the dried stems of *Tinospora crispa* in a male rat model of hepatic fibrosis caused by the hepatotoxin, thioacetamide.

**Materials and Methods:**

The extract was gavaged daily to the rats, at doses of 100 and 200 mg/kg along with thioacetamide at a dose of 200 mg/kg twice weekly. To assess the effectivity of extract, against thioacetamide, the activity of aminotransferases (alanine aminotransferase, aspartate aminotransferase), alkaline phosphatase (AP); and bilirubin were measured, together with morphological and histopathological indices in the liver of healthy and thioacetamide-treated rats.

**Results:**

A significant increase in the activity of liver enzymes, bilirubin and G-glutamyl transferase and gross and histopathological changes were determined. Although previous *in vitro* study established that this extract had strong antioxidant activity, this *in vivo* study establishes that this extract contains hepatotoxins whose identity may be quite different from those compounds with antioxidant properties.

**Conclusion:**

The study confirms that complete reliance on data obtained using *in vitro* methodologies may lead to erroneous conclusions pertaining to the safety of phytopharmaceuticals.

## Introduction

*Tinospora crispa;* is known by various vernacular names such as `akar seruntum` or `akar patawali` to the Malays. It is a climber plant belonging to the family of Menispermaceae. *T. crispa* is an indigenous plant and can be found distributed from the southwestern part of China to Southeast Asia, including Malaysia. Traditional folklore attributes various therapeutic uses to its stem for treatment of fever, jaundice, hyperglycemia,[1] hypertension, wounds, intestinal worms and skin infections. It is also used to treat tooth and stomach aches, cough, asthma and pleurisy.[2]

The whole plant contains a bitter principle, columbine, (2.22%) traces of an alkaloid and a glucoside. Three compounds, identified as *N*-cis-feruloyityramine, *N*-trans-feruloyltyramine and secoisolariciresinol, exhibiting antioxidant and radical scavenging properties toward carotene and 2,2-diphenyl-1-pierylhydrazyl (DPPH) radical, were isolated from the ch _2_cl _2_extract of stems of *T. crispa*.[3]

Two triterpenes are extracted from the stem of *T. crispa* namely cycloeucalenol and cycloeucalenone.[4] *T.* crispa stem contains: flavones O-glycosides (apigenine), picroretoside, berberine, palmatine, picroretine and resin. Flavonoids are naturally occurring polyphenolic compounds ubiquitously found in plants. The health benefits of flavonoids attributed to polyphenols is usually linked to two properties namely inhibition of certain enzymes such as xanthine oxidase and antioxidant activity.[5] They have long been recognized to possess anti-inflammatory, antioxidant, antiallergic, hepatoprotective, antithrombotic, antiviral and anticarcinogenic activities.[6] Based on the traditional uses of *T. crispa*, there has been a substantial increase in the sales of this part of the plant by certain pharmaceutical companies as capsules containing this natural product to treat fever, hypertension, as a diaphoretic tonic and antihyperglycemic agent. Furthermore, a study in rats, showed that 95% ethanol extract of *T. crispa* stem reduces blood sugar in alloxan-induced diabetic rats.[7]

Against this background, the present study was undertaken to assess the effect of ethanolic extract prepared from the dried stems of *T. crispa* in a rat model of hepatic fibrosis caused by (TAA). TAA is a hepatotoxin frequently used to induce hepatocellular injury and hepatic fibrosis in rats, and its prolonged administration causes the development of cirrhosis associated with an increased extent of lipid peroxidation.[8] These data were compared to identical data from rats, which were exposed to TAA only and a normal control group.

## Materials and Methods

### 

#### Plant material and preparation of its alcoholic extract

Dried and ground stems of *T. crispa* (obtained from Ethno Resources SDN. BHD) were weighed, homogenized in 95% ethanol at a ratio of 1:10 and left to macerate for 3 days at 25%C with occasional shaking and stirring. The mixture was then filtered and the resulting liquid was concentrated under reduced pressure at 45%C to obtain a dark gummy-green extract. The concentrated extracts were then frozen and finally lyophilized with freeze dryer, yielding the crude extract of the stems of *T. crispa*. The extract was then dissolved in 10% Tween-20 before administered orally to the animals.

#### Preparation of thioacetamide

Thioacetamide stock solution of 5 g/L was prepared by diluting the pure thioacetamide which was in the crystal form. The amount of thioacetamide needed was diluted in distilled water and stirred well until all the crystal thioacetamide was dissolved.

#### Experimental animals

Fifty male SD (Sprague Dawley) rats weighing 150-270 g at the beginning of the study were purchased from the animal house, Faculty of Medicine University of Malaya and Ethics No. PM/28/08/2009/MAA (R). They were kept in specially prepared cages at room temperature (23-32%C) with 12 hours light/12 hours dark photoperiod and 50-60% humidity in order to maintain normal circadian rhythm in the animal room. The rats were fed ad libitum with rodent food pellet, water was given through special dropper-tipped bottles, placed on the cages.

Rats were randomly divided into five groups. The animals were acclimatized under standard laboratory condition for a period of 2 weeks before the commencement of the experiment. One of the five groups of rats was designated as control group (Group 1) in which the rats (*n* = 8) were neither injected nor treated; Group 2 rats (*n* = 10) were gavaged daily with distilled water and were daily injected intraperitoneally with normal saline for 8 weeks. Group 3 rats (*n* = 12) were gavaged daily with distilled water and were injected intraperitoneally twice weekly with 200 mg/kg TAA for 8 weeks.[9] Group 4 rats (*n* = 10) were gavaged daily with 100 mg/kg of *T. crispa* extract with 200 mg/kg intraperitoneal injection twice weekly for 8 weeks. Group 5 rats (*n* = 10) were gavaged daily with 200 mg/kg of *T. crispa* extract with 200 mg/kg intraperitoneal injection twice weekly for 8 weeks.

The body weights of the animals were recorded starting from the day 0 and weekly throughout the experiment.

At the end of the 8 weeks, the rats were anesthetized by diethyl ether inhalation. Blood was drawn from jugular vein and the abdomen and thoracic cavity was opened. The liver was observed carefully for any gross changes.

#### Measuring body and relative liver weight

Spleens were isolated; and weighed. The livers were weighed; and liver: body weight ratio (Liver index) was calculated. Liver were excised into pieces, and were kept in isotonic formalin for determination of histological assessment.

#### Biochemical analysis

Blood was withdrawn through the jugular vein and collected into plain tube with activated gel for of liver function tests.

The sample was allowed to clot, centrifuged and the serum sample was sent to the Clinical Diagnosis Laboratories of the University Malaya Hospital to determine alanine aminotransferase (ALT), aspartate aminotransferase (AST), alkaline phosphatase levels (AP), bilirubin, G-Glutamyl transferase and total serum proteins using an autoanalyzer.

#### Histological evaluation of the liver

Sections of the randomly selected fixed liver specimens from each group were embedded in paraffin and processed for light microscopy by staining individual sections with hematoxylin-eosin stain.

#### Statistical analysis

The results are presented as mean±-standard error mean. The one-way ANOVA test with post-hoc test was used to analyse the data, with *P*<0.05 as the limit of significance.

## Results

The changes in the weight of rats among experimental groups after 8 weeks were found to be significant. Significant reduction (P<0.05) were observed in the body weight between the (G3, 4 and 5) in comparison to (G1-2) [Table 1] . This failure to thrive in (G3-5) was probably due to TAA and daily dosing with *T. crispa* extract. There was a significant (P<0.05) elevation in body weight in (G4-5) in comparison to G3.

**Table 1:** Changes in weight of rats in experimental groups after 8 weeks

*Groups*	*No. of animals (n)*	*Body weight (g)*	*Liver weight (g)*	*Liver index*
1	8	248.9987 ± 1.93078	5.0000 ± 0.07071	2.0200 ± 0.00567
2	10	260.5000 ± 1.21335	5.6000 ± 0.05627	2.1580 ± 0.02356
3	12	*179.3715 ± 1.29216	*7.1238 ± 0.06569	*3.9862 ± 0.00371
4	10	*#7.1238 ± 0.06569	*9.9000 ± 0.09489	*4.7700 ± 0.13314
5	10	*#213.9000 ± 1.17851	*22.3000 ± 0.37749	*5.3150 ± 0.05860

Data are presented as the mean ± standard error of mean.

* *P*<0.05 represent the significance of the difference of G3, 4 and 5 in comparison to G1 and 2;

# *P*<0.05 represent the significance of the difference of G4 and 5 in comparison to G3

Liver body weight ratios for (G3, 4 and 5) were significantly higher than those determined for (G1-2). No significant changes in the weight of the spleen for all groups of rats were noticed [Table 1] .

At the end of 8 weeks and as compared to control rats, TAA-treated rats had raised serum activities of ALT, AST, AP, bilirubin and G-Glutamyl transferase levels [Table 2].

**Table 2:** Biochemical results in experimental animals

*Biochemical results in experimental animals*							
*Groups*	*No. of animals (n)*	*ALT (IU/L)*	*AST (IU/L)*	*AP (IU/L)*	*Bilirubin (µmol/ L)*	*GGT (IU/L)*	*Alb.(g/L)*
1	8	43.7500 ± 0.70717	39.2500 ± 0.19554	86.7500 ± 0.19380	1 ± 0.04629	3.0000 ± 0.03651	29.3750 ± 0.70582
2	10	45.1000 ± 0.10541	34.3000 ± 0.33764	93.0000 ± 0.42531	1.1 ± 0.03651	3.1000 ± 0.05774	22.0000 ± 0.40825
3	12	*82.5556 ± 0.33500	*241.6667 ± 0.33660	*275.5556 ± 0.48760	*3 ± 0.22361	*12.0833 ± 0.10775	*10.9000 ± 0.20602
4	10	*97.5556 ± 0.18727	*247.4444 ± 0.25749	*318.7778 ± 0.44264	*3.5 ± 0.10801	*47.3000 ± 0.32455	*10.2222 ± 0.09793
5	10	*88.8889 ± 0.22374	*234.7778 ± 0.37447	*279.5556 ± 0.43239	*5 ± 0.18634	*41.900 ± 0.31833	*10.3333 ± 0.18637

Data are presented as the mean ± standard error of mean.

* *P*<0.05 represent the significance of the difference of G3, 4 and 5 in comparison to G1 and 2;

ALT indicates alanine aminotransferase; AST, aspartate aminotransferase; AP, alkaline phosphatase ; GGT, G-Glutamyl transferase.

Total serum protein was preserved in both G1 and G2 compared to TAA-treated groups (G3, 4 and 5). Serum albumin showed a marked decrease in comparison to serum globulin level in (G3, 4 and 5)[Table 1] . The most common reason for a low albumin is chronic liver failure caused by cirrhosis.

**Figure 1:**
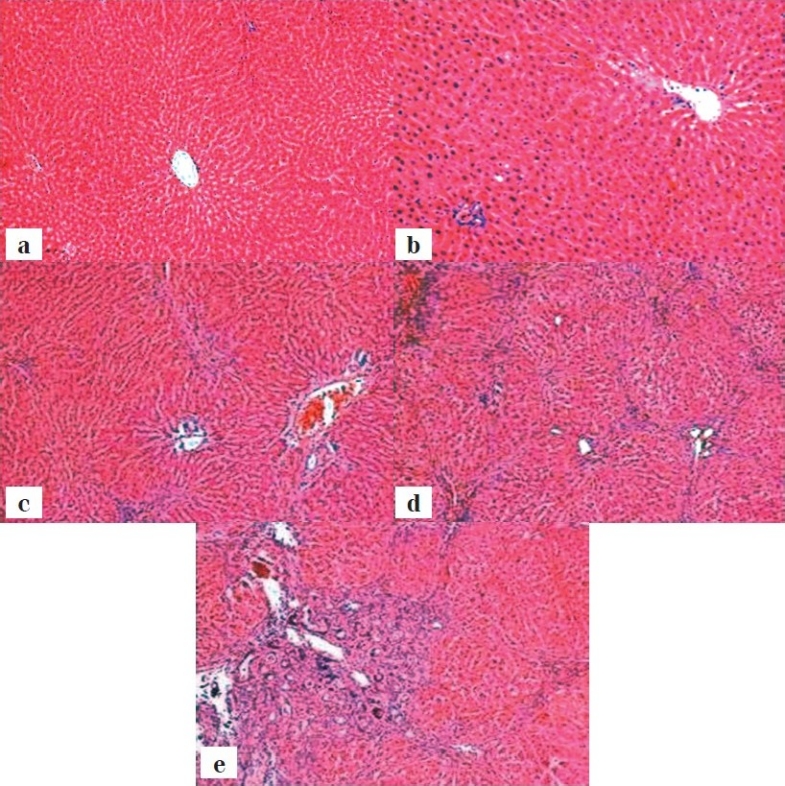
(a) Normal liver histology, (b) Liver histology of G2, (c) Liver histology of G3 showing the extension of fibrous septa in TAA- treated rats, (d) Thicker fibrous septa between the regenerative nodules in liver of rat with low-dose extract and TAA, (e) Thickest fibrous septa with proliferation of bile ducts. (H and E, ×10)

Microscopic examination revealed normal-appearing hepatic lobules and portal tracts in G1 and G2. In TAA group, hepatocytes degeneration, centrilobular necrosis of hepatocytes and inflammatory cell infiltration containing lymphocytes and mononuclear cells were observed and were aggravated by *T. crispa* extract, even at the lower dose [Figure 1].

## Discussion

Liver cirrhosis, a critical stage in chronic liver diseases with high morbidity and mortality, may be caused by viral infection, tissue-immune-mediated damage, toxic agents, obstructive jaundice, gene abnormalities, or alcohol and non-alcohol steatohepatitis.[10] One of the major function of the liver is detoxification of xenobiotics and toxins.[11] In many cases reactive oxygen species are produced during detoxification.[12]

TAA is a potent hepatotoxic agent. Some investigators have reported that oxidative stress may play an important role in TAA-induced acute liver injury.[8 13] This is evidenced by histopathological analysis and biochemical parameters (ALT, AST, AP, G-Glutamyl transferase and bilirubin) in plasma. However, the mechanism of oxidative stress in TAA hepatotoxicity is still unclear. TAAS-oxide and TAAS-dioxide are the main very reactive compounds produced during cytochrome-P450- mediated oxidation of TAA, besides free radicals.[14] These free radicals induce apoptosis and necrosis in liver cells.[15] Accordingly, the present study was undertaken to assess the effect of *T. crispa* in rat model of chronic liver disease in order to confirm that this plant does indeed have a therapeutic benefit in liver disease. We found long-term administration of *T. crispa* extract in TAA-treated rats potentiated the hepatotoxic effect of TAA, increased liver enzymes (ALT, AST, AP), G-Glutamyl transferase and elevated bilirubin levels, with reduction of body weight in animals. Collectively, these data indicate that ethanolic extracts of *T. crispa* at doses used in the study are hepatotoxic. Furthermore, its hepatotoxic effects are seemingly dose-dependent.

We are only aware of one unpublished study in which the effect of *T. crispa* has been evaluated against rifambicine in animal model of liver disease. One of the obvious differences between our study and that against refambicin is the experimental design. In our study the rats were treated daily for 8 weeks whereas in the other study, *T. crispa* extract was used in the dose of 100 mg/kg and 200 mg/kg four times at 12 intervals with a single dose of hepatotoxin.

Our study and other previously reported studies showed that *T. crispa* has a high antioxidant and radical scavenging activity potential established *in vitro*[3 16] in addition to containing antioxidant flavonol glycosides (apigenine), picroretoside, berberine, palmatine, picroretine and resin. All these compounds are responsible for the bitter principle of the stems of *T. crispa* extract which is freely soluble in alcohol.[3] The stems of *T. crispa* contain apigenin which is best known for its ability to act as a powerful antioxidant. Berberine compound from *Berberis aristata* was studied for its possible hepato-protective action in rats. Therefore, it is not unreasonable to assume that the widespread use of *T. crispa* in traditional practice of medicine in South-eastern Asia countries as well as registration of the drug (stem) in the Thailand Pharmacopoeia can be attributed to these antioxidant phytochemicals present in this plant.

The results of this study confirm that this plant enhances the hepatotoxic effect of TAA. This effect is seemingly time-dependent because a single oral dose of the decoction was beneficial. This discrepancy obviously warrants further investigation. We know that the TAA, is a thionosulfur-containing compound endowed with liver damaging and carcinogenic activity. In rats, shortly after administration of TAA and within 24 hour, it undergoes an extensive oxidative metabolism to an acetamide which is easily excreted outside the body through the urine. Whereas acetamide is devoid of liver-necrotizing properties, thioacetamide-S-oxide is further metabolized at least in part, by cytochrome P-450 monooxygenases to further products, including a polar product which is thought to be the sulfene, thioacetamide-S-dioxide, a very reactive compound. The binding of such metabolite to tissue may be responsible for the production of hepatic necrosis.[8]

As we reported that this plant is an antioxidant, we anticipated that there was an interaction between the antioxidant and the mixed function oxidase system of the TAA, which slows down or inhibits the oxidase enzymes of the metabolic process of TAA, resulting in accumulative toxicity of thioacetamide S, S-dioxide in the body, in addition to the delay in the transformation of thioacetamide S, S-dioxide to the end product (acetate) which might take more than 24 hr[8] in order to be excreted. Accordingly, the hepatotoxic action of TAA was more obvious in G4 and G5 than in G3.

Therefore, one can conclude that ethanolic extracts of *T. crispa* are hepatotoxic. Furthermore, we are unable to demonstrate exactly that this plant does indeed possess an antioxidant action *in vivo.* However, these data do not eliminate the possibility that the hepatotoxic action of *T. crispa* overwhelmed, thereby masking any antioxidant activity.

Although the *in vitro* study established that the ethanolic extract of *T. crispa* possessed bioactive compounds with antioxidant properties, the same study also hinted the plant had cytotoxic potential.[16] This *in vivo* study confirms the presence of hepatotoxins in the plant whose identity may be quite different from those compounds with antioxidant properties. Hence, this data highlights an important principle while conducting research into the supposed beneficial actions of medicinal plants. Our observations regarding using both *in vitro* and *in vivo* techniques indicate the importance of combined approach. Data obtained from *in vitro* experiments is useful for identifying a mechanism of action of plant extracts and giving an indication of their cytotoxic potential. The results of this study suggest that complementing *in vitro* experiments with those involving animals are essential steps in establishing the actual safety of medicinal plants and validating the supposed mechanism of action. Furthermore, these data confirm that complete reliance on data using *in vitro* methodologies alone may lead to erroneous conclusions about safety of phytochemicals.

## Conclusion

Ethanolic extracts prepared from the stems of *T. crispa* are commonly used to treat fever, diabetes and jaundice in traditional medicine in Malaysia, Thailand and Philippines. The results of this study demonstrate the hepatotoxicity of TAA is potentiated by the plant extract when administered daily for 8 weeks. This study also emphasizes the usefulness of a dual strategy involving *in vitro* and *in vivo* experiments on medicinal plants commonly used in traditional medicine, whose safety has not been yet been established.
